# The Sensitivity of Genetic Connectivity Measures to Unsampled and Under-Sampled Sites

**DOI:** 10.1371/journal.pone.0056204

**Published:** 2013-02-08

**Authors:** Erin L. Koen, Jeff Bowman, Colin J. Garroway, Paul J. Wilson

**Affiliations:** 1 Environmental and Life Sciences, Trent University, Peterborough, Ontario, Canada; 2 Wildlife Research and Development Section, Ontario Ministry of Natural Resources, Peterborough, Ontario, Canada; 3 Department of Zoology, Edward Grey Institute, University of Oxford, Oxford, United Kingdom; 4 Department of Biology, Trent University, Peterborough, Ontario, Canada; Lund University, Sweden

## Abstract

Landscape genetic analyses assess the influence of landscape structure on genetic differentiation. It is rarely possible to collect genetic samples from all individuals on the landscape and thus it is important to assess the sensitivity of landscape genetic analyses to the effects of unsampled and under-sampled sites. Network-based measures of genetic distance, such as conditional genetic distance (*cGD*), might be particularly sensitive to sampling intensity because pairwise estimates are relative to the entire network. We addressed this question by subsampling microsatellite data from two empirical datasets. We found that pairwise estimates of *cGD* were sensitive to both unsampled and under-sampled sites, and *F_ST_*, *D_est_*, and *d_eucl_* were more sensitive to under-sampled than unsampled sites. We found that the rank order of *cGD* was also sensitive to unsampled and under-sampled sites, but not enough to affect the outcome of Mantel tests for isolation by distance. We simulated isolation by resistance and found that although *cGD* estimates were sensitive to unsampled sites, by increasing the number of sites sampled the accuracy of conclusions drawn from landscape genetic analyses increased, a feature that is not possible with pairwise estimates of genetic differentiation such as *F_ST_*, *D_est_*, and *d_eucl_*. We suggest that users of *cGD* assess the sensitivity of this measure by subsampling within their own network and use caution when making extrapolations beyond their sampled network.

## Introduction

Genetic connectivity and gene flow are important for maintaining healthy populations of plants and animals; populations that exchange genes with other populations maintain or increase their genetic diversity and thus decrease their risk of extirpation [1]. It is therefore an important research goal to estimate gene flow and the habitat features and configurations that both promote and impede it, so that the effects of landscape structure on gene flow can be estimated. Assessing the relationship between genetic connectivity and landscape structure is a central goal in the field of landscape genetics [2], [3].

It is rarely possible to sample all individuals of the species or population of interest. Some areas may be logistically difficult to access, the researcher may be unaware of the existence of particular populations, it may be difficult to obtain samples from low-density populations, or it may not be financially feasible to genotype all of the collected samples. One challenge for landscape geneticists is thus to determine the appropriate spatial sampling scheme for collecting genetic samples. Samples can be collected uniformly across space, with the goal of conducting individual-based analyses [4]–[7], or many samples can be collected from several discrete sites with the goal of conducting site-based analyses [8]–[11]. Where and how many samples are collected may influence the conclusions drawn from landscape genetic analyses. For example, Schwartz and McKelvey [12] showed that the choice of sampling protocol can influence conclusions about population clustering. It is possible that individuals or sites that are instrumental for driving gene flow across the landscape have not been sampled in a given study. Beerli [13] described a scenario whereby two sampled populations exchange few migrants, but the presence of a third, unsampled population that supplies the same alleles to the first two populations could result in an overestimate of migration between the two sampled populations. For individual-based analyses, Landguth et al. [14] found that the number of sampled individuals does not influence the power of landscape genetic analyses relative to the numbers of loci and alleles, but stressed the need for a similar investigation with site-based study designs (but see [15]). It should therefore be of interest to landscape geneticists to investigate explicitly the influence that unsampled and under-sampled sites have on estimates of genetic differentiation and gene flow.

A second challenge for landscape geneticists is the choice of a metric that best estimates genetic differentiation between pairs of individuals or pairs of sites. Landscape genetic studies use a variety of metrics to index the same property: the amount of gene flow between two populations relative to the gene flow between other pairs of populations. For example, Hokit et al. [16] compared pairwise estimates of *F_ST_* to the least cost path (LCP) among sampling sites, whereas Lange et al. [17] and Dyer et al. [18] compared pairwise estimates of *D_est_* [19] and conditional genetic distance (*cGD*) [20], respectively, to the LCP among sampling sites. Unsampled and under-sampled sites might differentially affect these measures of genetic distance.

*F_ST_* [21] is a widely used measure of genetic fixation, calculated as a ratio of the variance in allele frequencies among populations to the overall variance. *D_est_* [19] is a measure of relative genetic differentiation between populations that predictably varies between 0 (no differentiation) and 1 (complete differentiation). *d_eucl_* [22] is akin to Rogers distance [23]; it is the straight-line distance between nodes based on allele frequencies of populations plotted in multivariate space [22]. Dyer and Nason [20] introduced *cGD* as a metric describing genetic differentiation between sampling sites based on network analyses. Conditional genetic distance represents the relative strength of the genetic covariance between sampled sites. Sites are represented by nodes in a network, and genetic differentiation is represented by edges in a network. Node centroids are defined by the mean of individuals at that site across alleles in multidimensional space. A saturated network of inter-population covariances, with edges connecting all nodes, is then pruned based on conditional independence, such that edges that do not contribute to the overall genetic covariance structure are removed. Conditional genetic distance between sites is then estimated as the shortest path through the pruned network. Dyer et al. [18] showed that *cGD* is a more powerful estimate of genetic differentiation than pairwise genetic distance estimates, such as *F_ST_*, in a landscape genetic context. This is because *cGD* considers the genetic information simultaneously from all sites and is thus dependent on which sites are included in the network. Because *cGD* estimates are relative to other connections within the network, they are likely sensitive to unsampled or under-sampled sites.

Concern over the effect of missing nodes on network parameters is not new: several studies have examined its effect on network attributes such as degree, clustering coefficient, path length, and betweenness in social networks [24]–[27]. Recently, Naujokaitis-Lewis et al. [28] investigated the sensitivity of genetic networks to unsampled nodes and found an effect of both sampling intensity and network algorithm (i.e., saturated, Gabriel, or minimum spanning tree). Similarly, Garroway et al. [29] assessed the resiliency of a population graph [20] to missing nodes. They found that the path length of their network changed little with the removal of the most connected nodes. Although these studies are useful in describing the effects of missing nodes on the global structure of networks (i.e., features that can only be determined by examining the entire network, such as betweenness, closeness, degree distribution, and path length), we do not know the effect of missing nodes on edge weight (particularly *cGD* obtained from population graphs), which forms the basis of landscape genetic analyses.

We subsampled empirical data to assess: (1) the sensitivity of *cGD* and other genetic distance estimators (*F_ST_*, *D_est_*, and *d_eucl_*) to unsampled and under-sampled sites, and how that sensitivity is influenced by both genetic structure and the connectivity of the unsampled or under-sampled sites; (2) the sensitivity of the rank order of pairwise *cGD* to unsampled or under-sampled sites; and (3) the effect that the change in rank order of *cGD* has on the outcome of landscape genetic analyses (i.e., Mantel tests). Finally, we used simulated data to demonstrate the influence that unsampled sites may have on our ability to detect isolation by resistance using *cGD*. To summarize, in part (1) we compared the relative error of *cGD* to *F_ST_*, *D_est_*, and *d_eucl_*. In parts (2) and (3), we focused on assessing the sensitivity of *cGD* to unsampled and under-sampled sites.

## Methods

### Data

**Figure 1:**
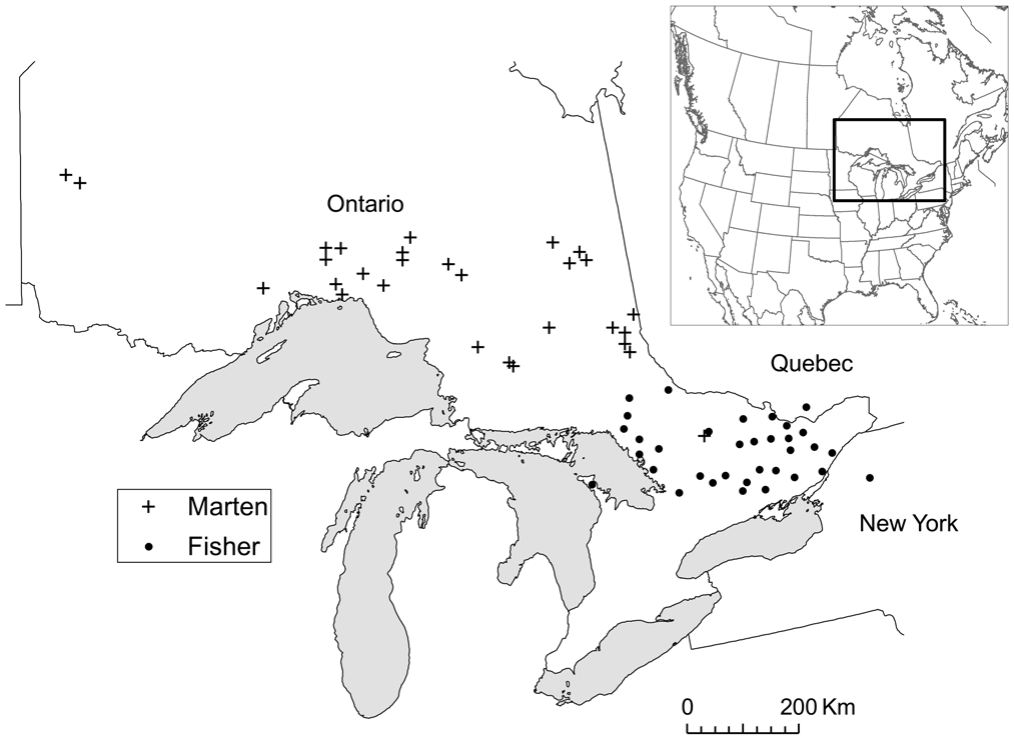
Locations of American marten (*Martes americana*; n = 29) and fisher (*M. pennanti*; n = 34) sampling sites. Inset shows location of Ontario and Quebec, Canada, and New York, USA, within central North America.

We used empirical microsatellite data from American martens (*Martes Americana*) and fishers (*Martes pennanti*) sampled in Ontario, Canada (Fig. 1) to test the effects of unsampled and under-sampled sites on 4 different genetic distance measures: *cGD*, *F_ST_*, *D_est_*, and *d_eucl_*. We used two different datasets because they varied in the extent of genetic clustering. The marten dataset contained 653 individual martens sampled at 29 sites and genotyped at 12 microsatellite loci [30]. There were 11–47 individuals sampled per site (average = 22.5, SD = 5.9) between 2004 and 2005. Previous work on this dataset revealed one genetic cluster (*K* = 1) [30]. The fisher dataset contained 772 individuals sampled at 34 sites across Ontario, northern New York State, USA, and southern Quebec, Canada, between 2000 and 2003 [8], [29], [31]. There were 7–48 individuals sampled per site (average = 21.2, SD = 7.1). The samples were genotyped at 16 microsatellite loci [8]. Previous work with these data suggested 5 genetic clusters (*K* = 5), with 5–11 sites per cluster [8], [29].

### Software

We used gstudio 0.6 [22] in R [32] to build genetic networks and estimate the network properties *cGD* and *d_eucl_* [18]. We estimated pairwise *F_ST_* [21] with software GENEPOP web version 4.0.10 [33]. We used SMOGD 1.2.5 [34] to estimate pairwise *D_est_* [19].

### Unsampled sites

We used the marten and fisher data to simulate studies where the population has not been fully sampled. For both species, populations were continuously distributed across the study areas, and we sampled individuals at discrete sites. For the purposes of our study, we assumed that the full dataset of 29 sites for marten and 34 sites for fisher provided a true measure of gene flow for each species; we considered all pairwise calculations of subsampled data relative to this “true” measure. It is likely that these full networks were, in fact, under-sampled representations of the true marten and fisher populations in Ontario, but for this study we assume that the full networks represented true gene flow.

We followed three steps to assess the response of gene flow estimates to unsampled sites: (1) we removed half of the sites (14 from the marten dataset, and 17 from the fisher dataset), leaving 15 and 17 sites in the marten and fisher datasets, respectively, and calculated pairwise *cGD*, *F_ST_*, *D_est_*, and *d_eucl_* for those remaining 15 or 17 sites; (2) we added one site to the network, and recalculated the pairwise *cGD*, *F_ST_*_,_ *D_est_*, and *d_eucl_* for the original 15 or 17 sites; (3) we repeated step two until we had included all 29 (marten) or 34 (fisher) sites; we considered this to be the full (true) dataset, although at each step we only recorded pairwise measures between the initial 15 (marten) or 17 (fisher) sites. By using pairwise estimates between the 15 or 17 sites only to compare mean genetic distance across iterations, we were able to hold sample size constant. We repeated this set of steps twice: in the first experiment (termed “least connected”), we retained the 15 (marten) or 17 (fisher) most connected sites (i.e., sites with the largest network eigenvector centrality), and the sites that we added back into the analysis, one site at a time, were the least connected sites (in the order of most to least connected). In the second experiment (termed “most connected”), we retained the 15 (marten) or 17 (fisher) least connected sites, and added the most connected sites into the analysis, one site for each iteration (in the order of least to most connected). In this way, we were able to assess the importance of an unsampled node's connectedness to measures of genetic connectivity in the rest of the network.

Eigenvector centrality is a node-based measure of the connectedness of a particular node and the nodes that are connected to it. It is a measure of a node's importance in a network as a function of how connected its neighbours are. We calculated eigenvector centrality with the software GeneticStudio [35] and ranked sites based on eigenvector centrality to identify which sites to remove (i.e., to identify the most or least connected sites). We chose eigenvector centrality over other measures of a node's importance, such as its degree or betweenness, because it distinguishes between nodes with the same degree that are connected to well-connected (or less well-connected) nodes.

### Under-sampled sites

We used microsatellite data from martens and fishers to simulate a study where the sites have been under-sampled. Once again, we considered the full dataset of 653 martens (at 29 sites) and 722 fishers (at 34 sites) to be the true measure of gene flow for each species, to which we compared all genetic distance estimates from the subsampled data.

We assessed the effect of under-sampled sites on pairwise measures of genetic distance by following three steps: (1) we removed half of the individuals from each site, with the exception of site 2 for the marten dataset and site 33 for the fisher dataset (see below), and calculated *cGD*, *F_ST_*, *D_est_*, and *d_eucl_*; (2) we added one individual back to each site, and recalculated *cGD*, *F_ST_*, *D_est_*, and *d_eucl_*; (3) we repeated step two until we had included all individuals. We repeated the series of steps 1–3 twice: in the first experiment (termed “rare”), the individuals that we removed in step 1 had rare genotypes relative to the entire sample, and we added those individuals back into the network in order of least to most rare. In the second experiment (termed “common”), the individuals that we removed in step one had common genotypes relative to the entire sample, and we added individuals back into the network in order of least to most common.

We identified individuals with rare genotypes by conducting a principal component analysis on allele frequencies for all individuals with the Adegenet (1.3) [36] and Ade4 (1.4–14) [37] packages for R. We extracted the scores for the first component for each individual; the extreme positive and extreme negative scores represent individuals with rare genotypes (with respect to the first component) relative to individuals with scores close to zero. We then sorted individuals by site, and removed half of the individuals with the most extreme positive or negative scores (or scores closest to zero for the second simulation) for each site in step one.

For each iteration, we added 1 individual back to each site. The number of individuals per site varied between 11 and 47; this meant that not every site received an additional individual at each iteration. For example, site 27 of the marten dataset had only 11 individuals, therefore we retained six individuals and added one individual to that site for the first five iterations only; iterations 6–14 of the marten dataset did not include an additional individual at site 27. Site 2 of the marten dataset had 47 individuals (the next most well-sampled sites were sites 1 and 18 with 27 individuals each). Rather than remove one half of the individuals from site 2, we removed 13, which resulted in 13 iterations, such that we added one individual to sites one, two, and 18 at each of the 13 iterations. Site 33 of the fisher dataset had 48 individuals; we removed 17 of these individuals, resulting in 17 iterations (rather than 24 iterations had we removed one half of the individuals from site 33).

### Relative Error

For both datasets, and for both unsampled and under-sampled sites, we calculated the absolute relative error between the mean of the pairwise genetic distance estimate at each iteration and the ‘true’ estimate (i.e., the estimate based on all data):
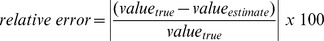



We compared mean estimates of relative error with Cohen's effect size *d* (the difference between group means, divided by the pooled standard deviation), using Cohen's general guidelines that *d* = 0.2 is a small effect, *d* = 0.5 is a medium effect, and *d* = 0.8 is a large effect [38]. We also used two-sample permutation tests (9999 randomizations) with DAAG (1.12) [39] in R to compare mean estimates of relative error between genetic distance measures, genetic structure (i.e., marten (one genetic cluster) or fisher (5 genetic clusters)), or experiment (i.e., most or least connected sites, or common or rare individuals added to the dataset at each iteration); we made specific comparisons depending on the question that we asked, rather than making all possible comparisons, and used α = 0.05 [40].

### Unsampled vs. under-sampled sites

We used effect size and two-sample permutation tests (9999 randomizations with DAAG in R) to assess the relative sensitivity of *cGD* to unsampled versus under-sampled sites. We pooled data over experiment (most and least connected sites) for the unsampled scenario, and over experiment (common and rare individuals) for the under-sampled scenario, and compared mean absolute relative error between unsampled and under-sampled scenarios separately for the marten and fisher datasets.

### Overview of study design

For our experiment assessing the effects of unsampled sites, we removed half of the sites from the full dataset. All calculations of relative error were based on the remaining sites. In the first iteration, we calculated our summary statistic (*cGD*, *F_ST_*, *D_est_*, or *d_eucl_*) for those remaining sites. We then calculated relative error by comparing these estimated summary statistics to estimates of the same statistics for the full dataset. For the second iteration, we added one site, and recalculated the summary statistic for the original set of remaining sites. In this experiment, we expected summary statistics of pairwise estimates (*F_ST_*, *D_est_*, and *d_eucl_*) to have relative errors of zero, because the inclusion of other sites in the dataset should not influence these pairwise statistics. However, we expected that the inclusion of other sites in a genetic network should influence *cGD*, even if the exact same pairs of sites are compared at each iteration, because calculations of *cGD* are based on the genetic covariance of all sites present in the dataset.

For our experiment assessing the effects of under-sampled sites, we removed half of the individuals from each site. At each subsampling iteration, we added one individual per site, and re-calculated the summary statistics. Our calculations of relative error compared all sites (29 for marten, 34 for fisher), with the subsampled iterations having fewer individuals per site, and the full dataset including all individuals per site. We expected all of the summary statistics that we tested to be sensitive to under-sampled sites.

### Sensitivity of between-node cGD rank

We were interested in the effect of unsampled and under-sampled sites on the rank order of pairwise *cGD* values within the network (i.e., whether pairs of sites with high relative *cGD* remained relatively high when new sites or individuals at each site were added to the network). For each iteration of the network, we used Spearman's rank correlation (ρ) to compare the rank order of the pairs to the ‘true’ network. If there was no effect of unsampled or under-sampled sites on the relative ranking of *cGD*, we expected a high Spearman's ρ (i.e., few deviations in the rank value between each iteration and the full network). We compared the mean (across iterations) Spearman's ρ between marten and fisher datasets and between experiments (least vs. most connected sites, or common vs. rare individuals added at each iteration) by considering both effect size and two-sample permutation tests (9999 randomizations with DAAG in R).

We used effect size and two-sample permutation tests to assess the relative sensitivity of the rank order of *cGD* to unsampled versus under-sampled sites. We pooled data over experiment (most and least connected sites) for the unsampled scenario, and over experiment (common and rare individuals) for the under-sampled scenario, and compared Spearman's ρ between unsampled and under-sampled scenarios separately for the marten and fisher datasets.

### Effect of unsampled and under-sampled sites on landscape genetic analyses

We were interested in assessing how unsampled or under-sampled sites affected our ability to detect landscape genetic relationships based on *cGD*. Both the marten [30] and fisher [8] datasets showed an isolation by distance pattern. We calculated the Mantel correlation coefficient *r* [41] of *cGD* and log Euclidean distance for each iteration with the Ecodist package (1.2.7) [42] in R (9999 permutations). We used bootstrapping in Ecodist to calculate 95% confidence intervals for the full dataset (10,000 iterations, sampling 90% of the data without replacement [42]). If there was no effect of unsampled or under-sampled sites on landscape genetics relationships, we expected to see a significant (α = 0.05) Mantel *r* statistic for each iteration, as we did for the full datasets. We compared the absolute mean (across iterations) relative error of the Mantel *r* statistic between datasets and simulations with effect sizes and two-sample permutation tests (9999 randomizations).

### Isolation-by-resistance simulation

**Figure 2:**
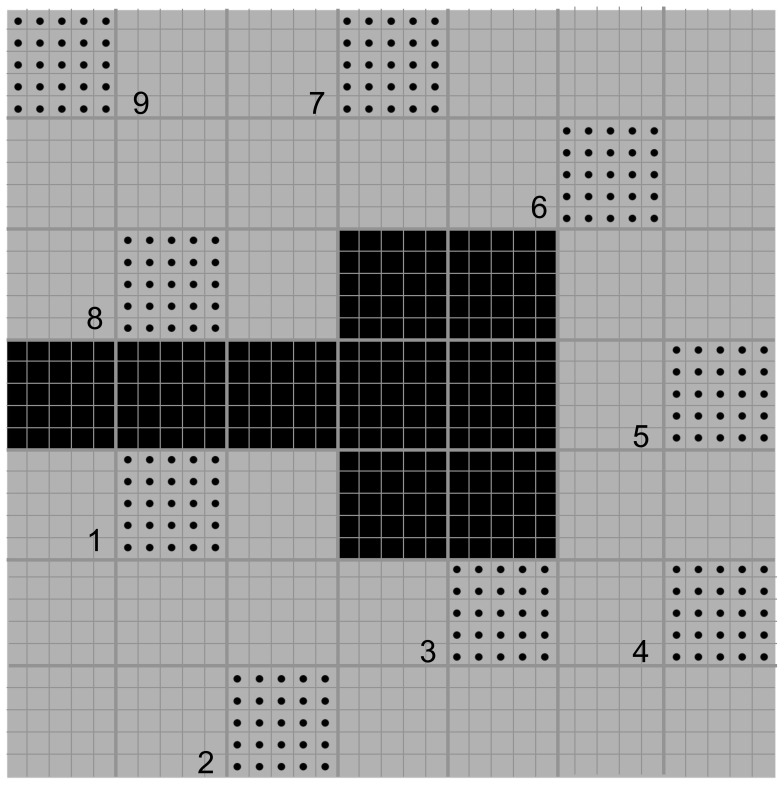
The cost surface used in the simulation of isolation by resistance. Grey cells represent low cost and black cells represent a barrier. We simulated gene flow between black dots for 250 generations. Numbers represent sites. Juveniles can disperse up to two sites away from their natal site. Unsampled populations are sites 4, 5, and 6; they contribute to gene flow but are not included in the calculation of genetic differentiation.

We used a simulation to visualize the influence of unsampled sites on estimates of *cGD* across a simple cost surface (Fig. 2). Our cost surface consisted of 49 cells (5×5 units within each cell); nine cells acted as a complete barrier to movement (black cells) whereas we assigned the remaining cells a low cost of one. We populated nine cells in the landscape with 50 individuals at each cell (one male and one female at each of 25 units in a cell, such that each pair of individuals in a cell was 1 unit apart), for a total of 450 individuals. Each individual was randomly assigned a genotype of 15 loci with 10 possible alleles per locus, with a k-allele mutation rate of 0.0005. We used program CDPOP version 1.2.05 [43] to simulate dispersal and mating between individuals at all nine sites for 250 non-overlapping generations. At each generation, 50% of the adults died and each mated pair produced four offspring in an equal sex ratio. Individuals moved up to 3 units to mate (with replacement), and juveniles dispersed as a function of the inverse-square of cost with a maximum cost distance of 25 units; this allowed individuals to disperse no farther than to two sampled cells away from their natal site. We replicated this simulation 21 times. Our simulation parameters were similar to those used in other studies [14], [44], [45], [46].

We selected sites 4, 5, and 6 (Fig. 2) to be unsampled sites; these sites contributed to gene flow (i.e., they were included in the CDPOP simulation), but were not subsequently sampled (i.e., the genotypes of individuals from these sites did not contribute to our pairwise estimates of genetic distance). Thus, we calculated all pairwise estimates of *cGD* between the core sites (sites 1, 2, 3, 7, 8, and 9) only. To investigate the influence of these unsampled sites on pairwise *cGD* estimates, we recalculated *cGD* three times. In the first calculation, we included one of the unsampled sites (site 6) in the network, but calculated mean pairwise *cGD* for the 6 core sites only. In the second calculation, we included 2 of the unsampled sites (sites 4 and 6) in the network. In the third calculation, we included all 3 unsampled sites (sites 4, 5, and 6) in the network, and calculated *cGD* between the 6 core sites only. In this way, we were able to assess the influence of including these sites without altering sample size or study area extent.

We calculated Mantel *r* values using pairwise *cGD* estimates and the cost distance (log-transformed) between the core 6 sites with the package Ade4 (1.4–14) [37] in R (9999 permutations). We used Cohen's [38] effect size and two-sample permutation tests (9999 randomizations with DAAG in R) to compare mean (across all 21 replicate simulations) Mantel *r* values between estimates based on networks that included 1, 2, or all 3 of the previously unsampled sites.

## Results

We used 4 summary statistics to describe our full, not sub-sampled, datasets (Table 1).

**Table 1:** Summary of genetic datasets^1^ used in a study of genetic connectivity measures.

Summary statistic	Marten			Fisher		
	Mean	SD	Range	Mean	SD	Range
*F_ST_* 2	0.022	0.018	0–0.102	0.068	0.046	0–0.266
*D_est_* 3	0.019	0.022	0–0.125	0.073	0.068	0–0.349
*D_eucl_* 4	2.719	0.544	1.44–4.86	5.390	1.376	1.930–10.291
*cGD* 4	6.533	2.040	2.448–11.26	10.25	3.749	2.678–9.97

1 The marten (*Martes americana*) dataset (n = 653, sampled at 29 sites) can be described as one genetic cluster. The fisher (*M. pennanti*) dataset (n = 772, sampled at 34 sites) was comprised of five genetic clusters. Both marten and fisher datasets originated from samples collected in Ontario, Canada. The number of clusters was determined by program Structure [54]

2 Estimated with GENEPOP web version 4.0.10 [33]

3 Estimated with SMOGD 1.2.5 [34]

4 Estimated with gstudio 0.6 [22]

### Sensitivity of genetic distance measures to unsampled sites

**Figure 3:**
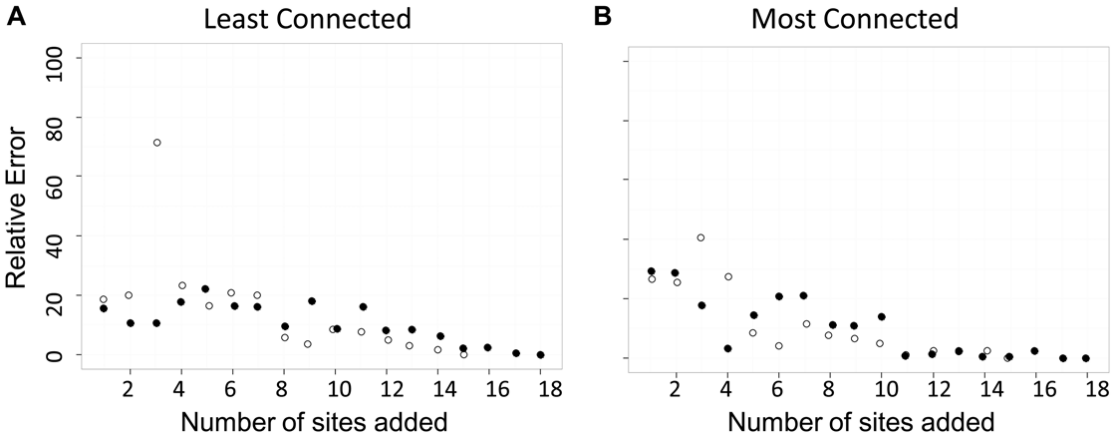
The effect of unsampled sites on conditional genetic distance (*cGD*) estimates. We represented the marten (*Martes americana*) dataset with hollow symbols and the fisher (*M. pennanti*) dataset with filled symbols. We have presented values as the absolute relative error (%) between the mean pairwise *cGD* estimate at each iteration and the *cGD* estimate for the full dataset (‘true’ measure). Each iteration represents the addition of one site to the analysis, where the first iteration has 15 (marten) or 17 (fisher) sites, and the last iteration has 29 (marten) or 34 (fisher) sites. In a), the sites that we added back into the analysis were the least connected, and in b), the sites that we added back into the analysis were the most connected (according to eigenvector centrality estimates).

We estimated the absolute relative error between the full set of data and datasets with fewer sampled sites (Fig. 3, Table 2). The average absolute relative error of *cGD*, across all iterations and all simulations of the effect of unsampled sites, was 12.3%. We found that *cGD* was more sensitive to the effects of unsampled sites than *F_ST_*, *D_est_*, and *d_eucl_*: not surprisingly, the mean absolute relative error was zero across all iterations for *F_ST_*, *D_est_*, and *d_eucl_*. The mean absolute relative error for *cGD* was significantly greater than zero (*P*<0.001 for all combinations of marten and fisher datasets and most and least connected sites removed) and the effect size was large (range *d* = 1.3–2.5).

**Table 2:** Absolute mean percent relative error (RE) of genetic distance estimates across iterations.

	Genetic measure	Dataset^1^	Units added^2^	Mean (RE)	SD
Unsampled	*cGD*	Marten	Least connected	16.05	17.70
			Most connected	12.22	12.48
		Fisher	Least connected	11.16	6.31
			Most connected	10.54	10.20
	*d_eucl_, D_est_, F_ST_*	Marten	Least connected	0	0
			Most connected	0	0
		Fisher	Least connected	0	0
			Most connected	0	0
Under-sampled	*cGD*	Marten	Common	31.71	30.36
			Rare	23.74	21.41
		Fisher	Common	32.95	35.26
			Rare	27.63	31.86
	*d_eucl_*	Marten	Common	6.95	6.65
			Rare	7.31	7.58
		Fisher	Common	8.04	8.37
			Rare	1.58	2.40
	*D_est_*	Marten	Common	15.91	12.94
			Rare	17.15	8.69
		Fisher	Common	11.62	10.03
			Rare	18.55	16.74
	*F_ST_*	Marten	Common	10.82	8.40
			Rare	5.49	4.67
		Fisher	Common	14.47	14.09
			Rare	9.40	8.38

1 The marten dataset (*K* = 1) is unclustered and the fisher dataset (*K* = 5) is clustered

2 The sites that we added back into the analysis were either the least or the most connected (according to eigenvector centrality estimates). The individuals that we added to each site had either common or rare genotypes (according to scores on the first principal component).

We did not find a difference in the sensitivity (mean absolute relative error) of *cGD* to unsampled sites that were strongly or weakly connected (marten dataset *d* = 0.250, *P* = 0.558; fisher dataset *d* = 0.073, *P* = 0.832). We also did not find a difference in the sensitivity of *cGD* to unsampled sites between the fisher or marten datasets (least connected sites removed, *d* = 0.368, *P* = 0.340; most connected sites removed, *d* = 0.147, *P* = 0.680).

### Sensitivity of genetic distance measures to under-sampled sites

**Figure 4:**
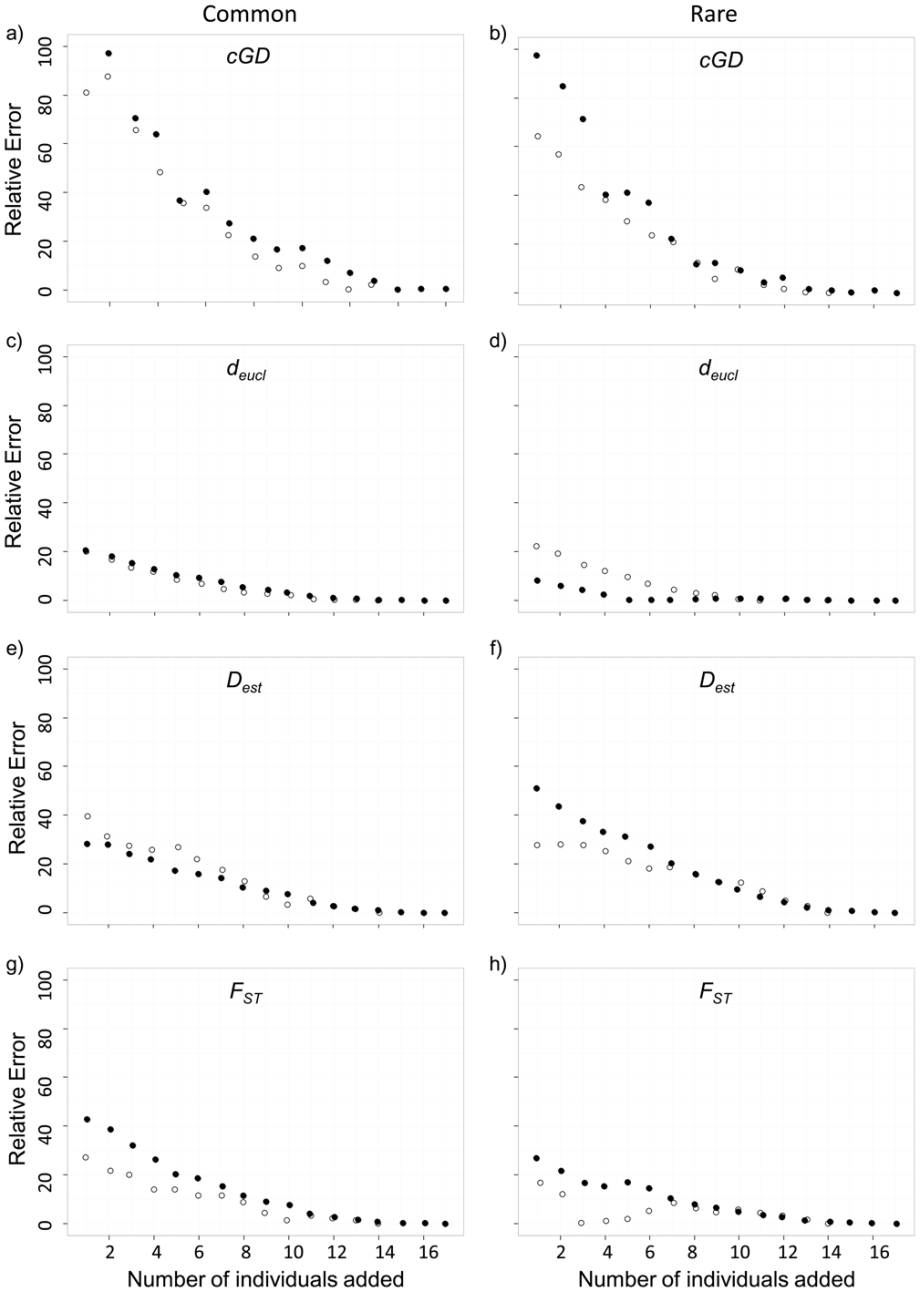
The effect of under-sampled sites on genetic distance estimates. We represented the marten (*Martes americana*) dataset with hollow symbols and the fisher (*M. pennanti*) dataset with filled symbols. We have presented values as the absolute relative error (%) between the mean pairwise genetic distance estimate at each iteration and the genetic distance estimate for the full dataset (‘true’ measure). Each iteration represents the addition of one individual to each site, such that in a, c, e, and g, the individuals added have common genotypes, and in b, d, f, and h, individuals added have rare genotypes (according to scores on the first principal component).

We found that mean relative error was higher for simulations with common individuals removed than with rare individuals removed when we measured genetic distance with *d_eucl_* for the fisher (*d* = 1.049, *P* = 0.0037) but not the marten (*d* = 0.050, *P* = 0.898) dataset (Fig. 4, Table 2). Mean relative error for the simulations with common individuals removed was higher than with rare individuals removed when we used *F_ST_* for the marten dataset (*d* = 0.784, *P* = 0.054) but not the fisher dataset (*d* = 0.436, *P* = 0.230). We found no difference in mean relative error between simulations with rare or common individuals removed from each site for *cGD* or *D_est_* (range *d* = 0.112–0.503).

The mean relative error was higher for the marten than the fisher dataset when we used *d_eucl_* as the measure of genetic distance and removed rare individuals from the simulations (*d* = 1.019, *P* = 0.005). Otherwise, we found no difference in mean relative error between marten and fisher datasets (range *d* = 0.038–0.578). We found general differences in mean relative error between genetic distance estimators (Table 3): *cGD* had higher relative error than both *d_eucl_* and *F_ST_,* and *D_est_* had higher relative error than *d_eucl_*.

**Table 3:** The difference in mean absolute relative error (%) between genetic distance estimators for under-sampled sites.

Comparison	Dataset^1^	Units added^2^	*P*-value^3^	Cohen's *d* 4
*cGD* vs. *d_eucl_*	Marten	Common	**0.006**	**1.127**
		Rare	**0.013**	**1.023**
	Fisher	Common	**0.004**	**0.927**
		Rare	**<0.001**	**1.153**
*cGD* vs. *D_est_*	Marten	Common	0.120	0.677
		Rare	0.317	0.403
	Fisher	Common	**0.019**	**0.823**
		Rare	0.324	0.357
*cGD* vs. *F_ST_*	Marten	Common	**0.025**	**0.938**
		Rare	**0.002**	**1.178**
	Fisher	Common	**0.048**	**0.688**
		Rare	**0.031**	**0.783**
*D_est_* vs. *d_eucl_*	Marten	Common	**0.016**	**0.871**
		Rare	**0.007**	**1.206**
	Fisher	Common	0.135	0.388
		Rare	**<0.001**	**1.419**
*F_ST_* vs. *d_eucl_*	Marten	Common	0.199	0.510
		Rare	0.468	−0.289
	Fisher	Common	0.062	0.554
		Rare	**<0.001**	**1.270**
*D_est_* vs. *F_ST_*	Marten	Common	0.148	0.467
		Rare	**<0.001**	**1.671**
	Fisher	Common	0.517	−0.233
		Rare	0.061	0.691

1 Marten data are not genetically clustered (*K* = 1); fisher data are genetically clustered (*K* = 5)

2 The sites that we added back into the analysis were either the least or the most connected (according to eigenvector centrality estimates). The individuals that we added to each site had either common or rare genotypes (according to scores on the first principal component)

3 Comparisons that are significantly different are in bold font at α = 0.05 based on permutation tests

4 Cohen's [38] effect size, where *d* = 0.2 is a small effect, *d* = 0.5 is a medium effect, and *d* = 0.8 is a large effect (large effects are in bold font)

When we considered the effect of under-sampled sites on the fisher dataset, in which individuals with common genotypes were added to the network at each iteration, the first, third, and sixth iterations resulted in a subdivided network. The result was two sub-networks that were not connected by an edge, and thus there were no pairwise estimates of *cGD* between sites in different sub-networks. This did not have an effect on pairwise estimates of *F_ST_*, *D_est_*, or *d_eucl_*.

### Unsampled vs. under-sampled sites

Mean absolute relative error of *cGD* was significantly greater for under-sampled than for unsampled sites (marten dataset *d* = 0.638, *P* = 0.0221; fisher dataset *d* = 0.804, *P* = 0.001). Similarly for *F_ST_*, *D_est_*, and *d_eucl_*, mean absolute relative error was greater for the under-sampled than for the unsampled sites (effect size *d* ranged from 0.698–1.62).

### Sensitivity of between-node cGD rank

**Figure 5:**
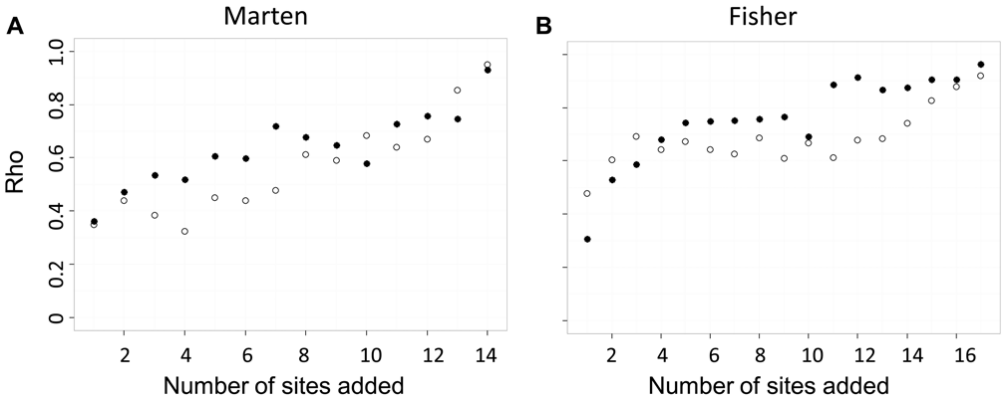
The effect of unsampled sites on the rank-order of *cGD* as measured by Spearman '**s ρ.** Higher values of ρ indicate fewer deviations in the rank order of *cGD* compared between each iteration and the true *cGD*. Each iteration represents the addition of one site to the analysis; hollow symbols represent the addition of the least connect sites and filled symbols represent the addition of the most connected sites.

The rank order of *cGD* changed at each iteration, and we used Spearman's ρ to quantify the deviation relative to the full dataset. Mean ρ ranged from 0.30 to 0.96 for iterations with unsampled sites (Fig. 5), where small values reflect high deviation. We found no difference in mean ρ between simulations with the most connected and the least connected sites added at each iteration (marten dataset *d* = 0.439, *P* = 0.249; fisher dataset *d* = 0.515, *P* = 0.149). The marten dataset (one genetic cluster) was more sensitive than the fisher dataset (five genetic clusters) to unsampled sites with respect to rank order of *cGD* (most connected sites removed *d* = 0.800, *P* = 0.039; least connected sites removed *d* = 0.816, *P* = 0.025).

**Figure 6:**
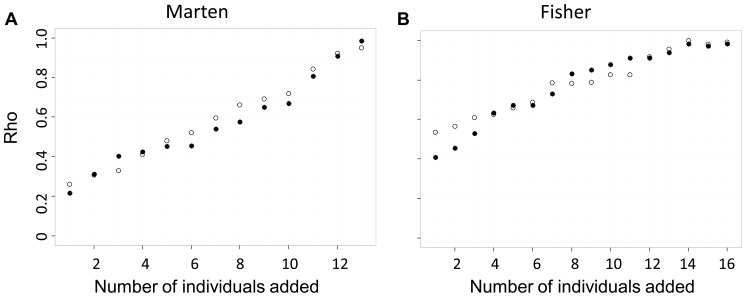
The effect of under-sampled sites on the rank-order of *cGD* as measured by Spearman '**s ρ.** Higher values of ρ indicate fewer deviations in the rank order of *cGD* compared between each iteration and the true *cGD*. Each iteration represents the addition of one individual to each site; hollow symbols represent the addition of individuals with common genotypes and filled symbols represent the addition of individuals with rare genotypes.

Mean ρ ranged from 0.22 to 0.99 for iterations with under-sampled sites (Fig. 6). We did not find a difference in the sensitivity of the rank order of *cGD* (i.e., mean ρ) to under-sampled sites that were missing individuals with common or rare genotypes (marten dataset *d* = 0.097, *P* = 0.804, fisher dataset *d* = 0.059, *P* = 0.865). We did, however, find an effect of genetic structure on the deviation of the rank order of *cGD* relative to the full dataset*:* the marten dataset was more sensitive (lower mean ρ) than the fisher dataset to under-sampled sites (common individuals removed *d* = 0.971, *P* = 0.0134; rare individuals removed *d* = 0.967, *P* = 0.015).

Mean Spearman's ρ for *cGD* did not differ between unsampled (pooled over most and least connected sites) and under-sampled (pooled over common and rare individuals) sites (marten dataset *d* = 0.0869, *P* = 0.744; fisher dataset *d* = 0.356, *P* = 0.153).

### Effect of unsampled and under-sampled sites on landscape genetic analyses

**Figure 7:**
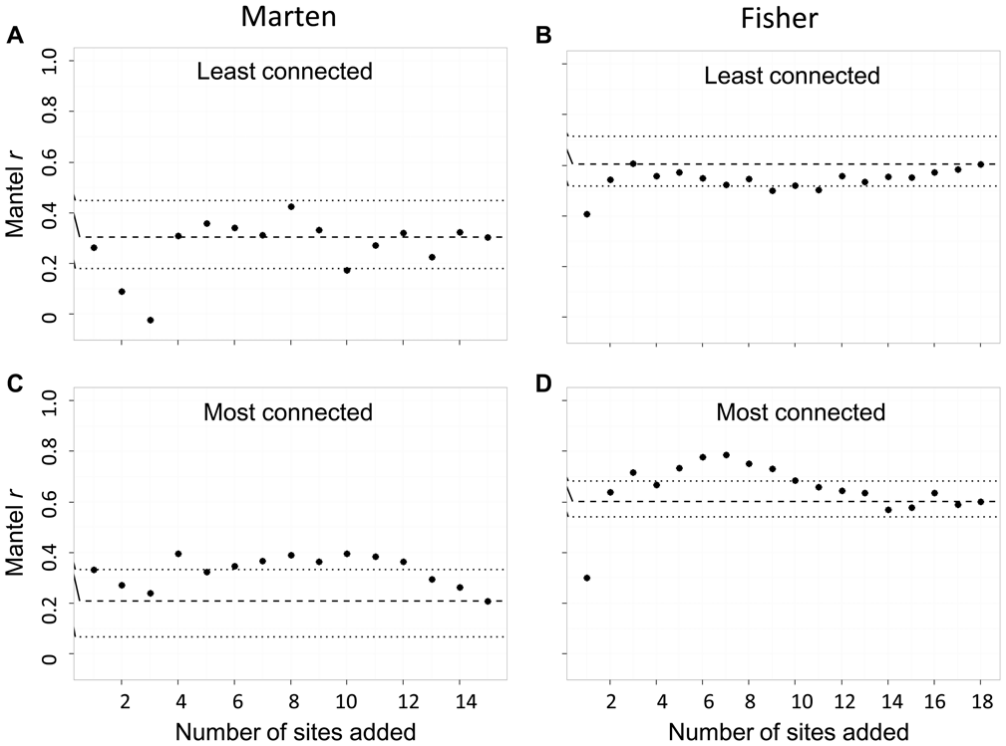
The effect of unsampled sites on Mantel *r* statistics testing isolation by distance. Each iteration represents the addition of one site to the network, such that sites are the least connected (panels a and b) or the most connected (panels c and d), for both the marten (*Martes americana*) and fisher (*M. pennanti*) datasets. The dashed line is the Mantel *r* (95% CI) statistic for the full dataset. Isolation by distance is the relationship between *cGD* and log Euclidean distance.

We used Mantel tests to compare estimates of *cGD* to log Euclidean distance. We found that unsampled sites affected the relative error of Mantel *r*: absolute relative error ranged from 0.43 to 106.8% (Fig. 7). We found a larger effect of unsampled sites when the sites left unsampled were the most-connected relative to when they were the least connected (marten dataset *d* = 1.299, *P* = 0.0038; fisher dataset *d* = 0.491, *P* = 0.160). We also found a larger effect of unsampled sites on the relative error of Mantel *r* for the marten dataset relative to the fisher dataset (least connected sites removed *d* = 0.699, *P* = 0.031; most connected sites removed *d* = 2.317, *P*<0.001).

**Figure 8:**
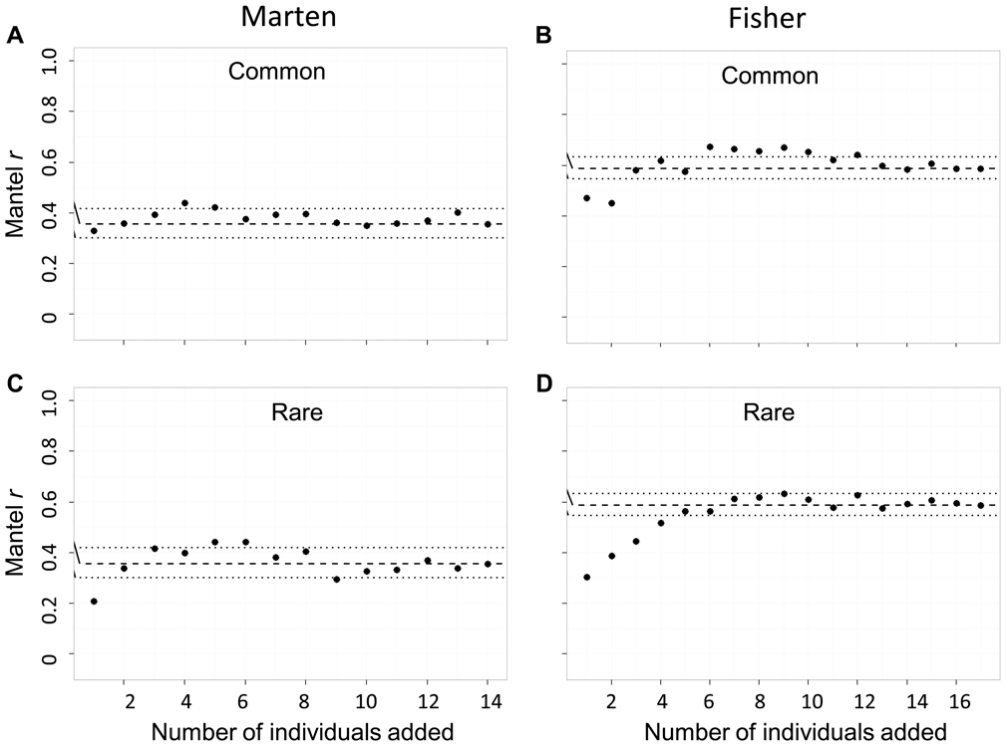
The effect of under-sampled sites on Mantel *r* statistics testing isolation by distance. Each iteration represents the addition of one individual to each site in the network, such that individuals have common genotypes (panels a and b) or rare genotypes (panels c and d), for both the marten (*Martes americana*) and fisher (*M. pennanti*) datasets. The dashed line is the Mantel *r* (95% CI) statistic for the full dataset. Isolation by distance is the relationship between *cGD* and log Euclidean distance.

We found that under-sampled sites also affected Mantel *r* estimates; absolute relative error of Mantel *r* ranged from 0.18 to 48.0% (Fig. 8). We found no difference in the effect of under-sampled sites on Mantel *r* between the marten and fisher datasets (common individuals removed *d* = 0.005, *P* = 0.990; rare individuals removed *d* = 0.331, *P* = 0.394). We found no difference in mean relative error between simulations with common or rare individuals left unsampled for both the marten (*d* = 0.606, *P* = 0.135) and fisher (*d* = 0.144, *P* = 0.691) datasets. Overall, mean relative error of Mantel *r* was larger for unsampled sites than for under-sampled sites for the marten dataset (*d* = 1.349, *P*<0.001) but not for the fisher dataset (*d* = 0.364, *P* = 0.147).

We found significant (α = 0.05) isolation by distance with simple Mantel tests for both full datasets (marten Mantel *r* = 0.355; fisher Mantel *r* = 0.586), and we found significant isolation by distance for every subsampled iteration except 3 of 14 iterations of the marten dataset where the least-connected sites were not sampled (Fig. 7a).

### Isolation-by-resistance simulation

**Figure 9:**
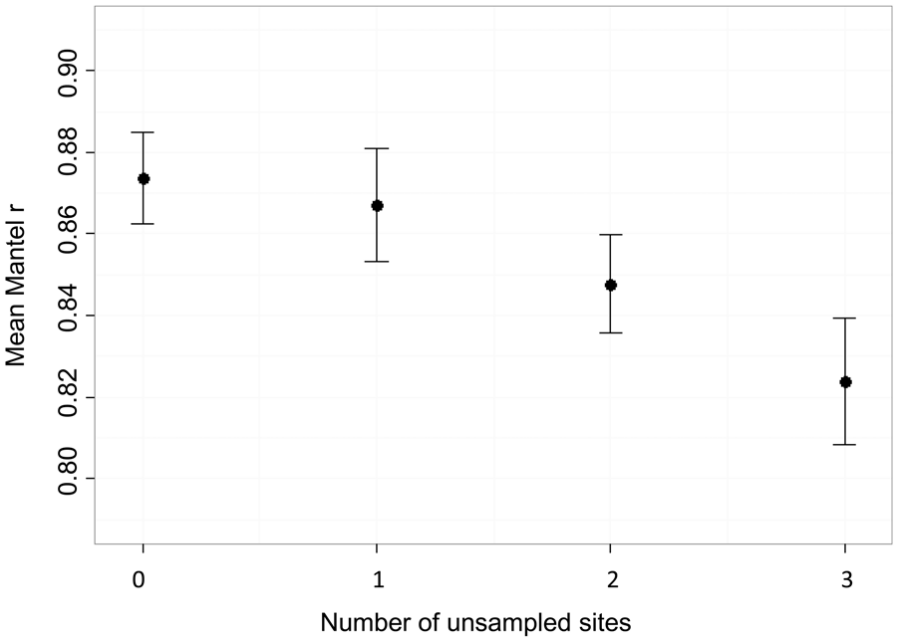
Mean (SE) Mantel *r* values across 21 replicates of simulated isolation by resistance. Mantel *r* values are based on conditional genetic distance (*cGD*) and the cost distance (log-transformed) between 6 pairs of sites. We calculated Mantel *r* values based on four estimates of *cGD*: when all nine sites were included in the network (0 unsampled populations), when 1 site (site 5) was not included in the network, when 2 sites (sites 4 and 5) were not included in the network, and when 3 sites (sites 4, 5, and 6) were not included in the network. We included only the 6 core sites (sites 1, 2, 3, 7, 8, and 9; see Figure 2) in all calculations of Mantel *r*.

Our simulation of isolation-by-resistance (Fig. 2) demonstrated the sensitivity of *cGD* and the network to unsampled sites because we knew for certain that isolation by resistance was driving gene flow. We found that increased sampling (i.e., including all nine populations in the network) resulted in more accurate estimates of *cGD* (Fig. 9): mean Mantel *r* was significantly larger, and thus closer to 1 which would imply perfect detection of the isolation by resistance process that we modeled, when we included sites 4, 5, and 6 than when we did not (*d* = 0.805, *P* = 0.011). We found that adding one site at a time to the network had only a small effect on mean Mantel *r* values (3 versus 2 unsampled sites, *d* = 0.376; 2 versus 1 unsampled site, *d* = 0.325; 1 versus 0 unsampled site, *d* = 0.114). For *F_ST_*, *D_est_*, and *d_eucl_*, Mantel *r* did not change as more sites were added (results not shown), implying that the accuracy of the estimate cannot increase with increased sampling.

## Discussion

We found that pairwise estimates of *cGD* were sensitive to both unsampled and under-sampled sites: absolute relative error was as high as 71.3 and 112.1%, respectively. Although all genetic estimators that we compared were sensitive to under-sampled sites, *cGD* was more sensitive than both *F_ST_* and *d_eucl_*. Similarly, with respect to unsampled sites, *cGD* was more sensitive than other measures of genetic differentiation such as *F_ST_*, *D_est_*, and *d_eucl_*. This is because these genetic differentiation measures are true pairwise measures; their calculations involve comparing the alleles present only at the pair of sites in question. Conversely, pairwise estimates of *cGD* are calculated based on the covariance of alleles present at all sites in the network. By its nature then, *cGD* will be sensitive to unsampled sites whereas *F_ST_*, *D_est_*, and *d_eucl_* will not. The interpretation of absolute relative error is challenging because we do not know how sensitive is too sensitive. For landscape genetic analyses, the answer may lie in whether this sensitivity leads to changes in the rank order of *cGD* estimates and conclusions about Mantel *r* or other test statistics.

The sensitivity of the rank order of *cGD* is important because it is the basis of current landscape genetic analyses: analyses of isolation by distance or isolation by resistance are rooted in correlations between pairwise estimates of genetic differentiation and pairwise estimates of Euclidean or cost distance. The rank order of pairwise *cGD* estimates determines the strength of this relationship. If pairs of sites with the smallest (or the largest) *cGD* estimates are closest to (or farthest from) one another in geographic space relative to the other sites in the study, the researcher might conclude a pattern of isolation by distance. If one or more sites were not sampled and by not sampling these sites the rank order of *cGD* estimates changed (i.e., pairs of sites separated by the largest geographic distances were no longer separated by largest genetic distances), then the researcher may not conclude isolation by distance, even though isolation by distance was indeed the process driving genetic structure. We found that unsampled and under-sampled sites caused the rank order of *cGD* to deviate relative to the full dataset. We quantified these deviations with Spearman's ρ, which we found to be as low as 0.30 and 0.22 for unsampled and under-sampled sites, respectively. Deviations from the full dataset of the rank order of *cGD* tended to become smaller as more sites or more individuals per site were included in the network (Figs 5 and 6), indicating that increased sampling can alleviate the concern of uncertain rank order of pairwise *cGD* estimates. We also found that the marten dataset (one genetic cluster) was more sensitive than the fisher dataset (five genetic clusters) to deviations in rank order of *cGD* due to both unsampled and under-sampled sites; researchers should be aware of this, particularly when their study system has minimal genetic structure. As with mean relative error of *cGD* estimates, it is difficult to determine the threshold value of Spearman's ρ at which one would conclude that deviations in rank order are too high. Ultimately, deviations in rank order are too high when the accuracy of the resulting test statistic decreases, leading to increased risk of type 1 error.

We found that Mantel *r* statistics were sensitive to unsampled and under-sampled sites: relative error was as high as 106.8 and 48%, respectively. We found Mantel *r* to be more sensitive to the effect of unsampled than under-sampled sites, especially when there was little genetic structure (marten data), and the sites that were not sampled were the most-connected sites. Our results suggest that landscape geneticists should invest in sampling more sites rather than more individuals per site, especially when there is minimal genetic structure, as this will increase the accuracy of Mantel *r* statistics. Landguth et al. [14] investigated statistical power in landscape genetic analyses using individual-based rather than site-based sampling designs; higher statistical power was obtained when effort was allocated toward increasing the number of microsatellite loci rather than number of individuals sampled. Hale et al. [15] found that the accuracy of allele frequencies in site-based analyses leveled off at 25–30 individuals sampled per site. To our knowledge, our study is the first to compare sensitivity of network-based *cGD* to unsampled and under-sampled sites.

Finally, we used permutations to test the statistical significance of Mantel *r* values. The full datasets showed significant isolation by distance, therefore we expected that if unsampled or under-sampled sites had no effect on the overall conclusions drawn from landscape genetic analyses, we would also find significant isolation by distance when we did not include all sites or all individuals in our *cGD* estimate. Overall, we found that unsampled and under-sampled sites did not affect our conclusions about isolation by distance, with the exception of 21% of the iterations simulating unsampled sites with the marten dataset. Despite the sensitivity of Mantel *r* values, unsampled or under-sampled sites might not cause landscape geneticists to draw incorrect conclusions about the processes driving gene flow in their studies. Users of *cGD* in a landscape genetic context should use caution, however, when their data have minimal genetic structure. The Mantel *r* values for the full datasets in our study were relatively large (0.355 and 0.586 for marten and fisher datasets, respectively). It is possible that the statistical significance of Mantel *r* will be more sensitive to unsampled and under-sampled sites for variables that correlate less well with genetic distance (i.e., smaller Mantel *r* for the full dataset). This, as well as the sensitivity of partial Mantel tests, should be addressed with future work [28]. Future work should also address the sensitivity of Mantel permutation tests to discern between significant patterns and random patterns [47].

Our study design allowed us to examine a number of variables with a range of values, making our results applicable to many landscape genetic studies using *cGD*. We considered clustered (fisher, *K* = 5) and unclustered (marten, *K* = 1) datasets, and we found that our marten dataset was more sensitive than the fisher dataset to the effects of both unsampled and under-sampled sites. Considering both clustered and unclustered datasets meant that we were considering a range of values when we looked at the effect of whether the unsampled sites were the most or least connected sites, or when the under-sampled sites were missing individuals with common or rare genotypes. We found that if unsampled sites were highly connected, Mantel *r* values were more sensitive than if unsampled sites were less connected, but this sensitivity is not strong enough to influence the conclusions drawn from statistical tests of isolation by distance. We found no difference between whether under-sampled sites were missing individuals with common or rare genotypes on the sensitivity of *cGD* or Mantel *r*. Our results should be applicable to other landscape genetic studies with less than 5 genetic clusters.

The fact that *cGD* is sensitive to unsampled and under-sampled sites is not surprising given that the metric is based on the relative covariance of the entire network. This sensitivity is not necessarily a negative trait, especially given that it does not, at least in our study, greatly affect the conclusions drawn from landscape genetic analyses. The effects of unsampled sites on landscape genetic analyses are difficult to study because we often use pairwise metrics of genetic differentiation, such as *F_ST_* or *D_est_*, that consider only the pair of sites in question. These pairwise metrics are not influenced by other sites, sampled or not, even if those other sites are having a marked influence on gene flow in the system. Pairwise estimates of *cGD*, on the other hand, are sensitive to other sites, and this sensitivity means that as more sites are sampled, the accuracy of *cGD* estimates increases and better reflects true gene flow. We showed this with our simulation (Figs 2, 9), where we knew that gene flow was governed by isolation by resistance. As we increased the number of sites that we sampled, the accuracy of our estimates of isolation by resistance improved. Because pairwise estimates such as *F_ST_* and *D_est_* are insensitive to the contribution to gene flow of other sites, the accuracy of these estimates cannot improve as sampling effort is increased. Thus, the sensitivity of *cGD* explains why it has the potential to be a powerful estimate of genetic distance [18].

The bottom line is whether *cGD* is too sensitive to unsampled and under-sampled sites to be a reliable estimate of genetic differentiation, especially given that few studies can claim to have sampled all sites that contribute to gene flow in their study system. It is our opinion that the pros (greater power to detect landscape genetic relationships, as per Dyer et al. [18]) outweigh the cons (the potential to draw incorrect conclusions from landscape genetic analyses), but users of *cGD* need to weigh the pros and cons for their own study systems.

We agree with Naujokaitis-Lewis et al. [28] that landscape geneticists should investigate sensitivity within their networks, recognizing that it is difficult to extrapolate within-network sensitivity to sites that were not sampled and for which we do not know the genotypes of individuals at those unsampled sites. A jackknifing procedure, where a random subset of the sites are included in the estimate of *cGD*, could be used in practice to estimate the variance of *cGD* due to unsampled sites. Conclusions about the effect of the landscape on differentiation derived from a genetic network should be extrapolated to areas and populations beyond the empirical network only with caution. We propose that *cGD* is appropriate for hypothesis testing in landscape genetics because the sensitivity of the estimate can result in more accurate conclusions. We do not know how accurately *cGD* predicts the relationship between genetic and landscape connectivity beyond the study area because estimates of *cGD* are conditional on which sites are included in the network. Subsequent, generalized hypotheses should only be extrapolated to a larger area with intentions of further validation. We suggest that users of *cGD* use caution when their study systems approach panmixia and, in general, should use *cGD* in conjunction with other pairwise measures of genetic distance [48]–[50]. Future work should investigate methods to mitigate the impacts of unsampled and under-sampled sites on landscape genetic analyses [13], [51]–[53].
